# Ablation of *Ggnbp2* impairs meiotic DNA double‐strand break repair during spermatogenesis in mice

**DOI:** 10.1111/jcmm.13751

**Published:** 2018-07-28

**Authors:** Kaimin Guo, Yan He, Lingyun Liu, Zuowen Liang, Xian Li, Lu Cai, Zi‐Jian Lan, Junmei Zhou, Hongliang Wang, Zhenmin Lei

**Affiliations:** ^1^ Department of Andrology The First Hospital of Jilin University Changchun China; ^2^ Department of OB/GYN University of Louisville School of Medicine Louisville KY USA; ^3^ Pediatrics Departments University of Louisville School of Medicine Louisville KY USA; ^4^ Division of Life Sciences and Center for Nutrigenomics & Applied Animal Nutrition Alltech Inc. Nicholasville KY USA; ^5^ Central Laboratory Shanghai Children's Hospital Shanghai Jiao Tong University Shanghai China; ^6^ Present address: Fujian Medical University Fuzhou Fujian China

**Keywords:** DSB repair, GGN1, GGNBP2, knockout, meiosis, spermatocyte, testis

## Abstract

Gametogenetin (GGN) binding protein 2 (GGNBP2) is a zinc finger protein expressed abundantly in spermatocytes and spermatids. We previously discovered that *Ggnbp2* resection caused metamorphotic defects during spermatid differentiation and resulted in an absence of mature spermatozoa in mice. However, whether GGNBP2 affects meiotic progression of spermatocytes remains to be established. In this study, flow cytometric analyses showed a decrease in haploid, while an increase in tetraploid spermatogenic cells in both 30‐ and 60‐day‐old *Ggnbp2* knockout testes. In spread spermatocyte nuclei, *Ggnbp2* loss increased DNA double‐strand breaks (DSB), compromised DSB repair and reduced crossovers. Further investigations demonstrated that GGNBP2 co‐immunoprecipitated with a testis‐enriched protein GGN1. Immunofluorescent staining revealed that both GGNBP2 and GGN1 had the same subcellular localizations in spermatocyte, spermatid and spermatozoa. *Ggnbp2* loss suppressed *Ggn* expression and nuclear accumulation. Furthermore, deletion of either *Ggnbp2* or *Ggn* in GC‐2spd cells inhibited their differentiation into haploid cells in vitro. Overexpression of *Ggnbp2* in *Ggnbp2* null but not in *Ggn* null GC‐2spd cells partially rescued the defect coinciding with a restoration of *Ggn* expression. Together, these data suggest that GGNBP2, likely mediated by its interaction with GGN1, plays a role in DSB repair during meiotic progression of spermatocytes.

## 1 INTRODUCTION

Spermatogenesis consists of three major stages which include proliferation and differentiation of spermatogonia, meiotic divisions of spermatocytes and metamorphosis of haploid spermatids, which are tightly controlled by a highly orchestrated expression of several thousand different genes.^1^, ^2^ Failure to faithfully and timely complete one or more of these stages leads to male infertility.

Gametogenetin binding protein 2 (GGNBP2) is an evolutionarily conserved zinc figure protein across humans, mice and rats.^3^, ^4^ The biological functions of GGNBP2 are diverse. It has been reported that GGNBP2 participates in the regulation of the G2/M transition of somatic cell cycle^5^, ^6^ and in the modulation of the differentiation of placental trophoblastic stem cells.^3^ A number of studies suggest that GGNBP2 may serve as a tumour suppressor as down‐regulation of GGNBP2 is found in several malignancies, such as laryngeal carcinoma,^7^, ^8^ breast cancer,^9^ ovarian cancer^10^, ^11^ and brain glioma.^12^ GGNBP2 deficiency promotes cell growth, drug resistance and tumorigenic progression.^10^, ^11^ GGNBP2 is highly expressed in the adult testis and its expression is tightly associated with the start of spermatogenesis.^4^, ^13^ Recently, we and others demonstrated that testicular GGNBP2 is indispensable for spermatogenesis.^4^, ^14^ *Ggnbp2* knockout (*Ggnbp2KO)* males are infertile and show extensive defects in spermatogenesis. We previously discovered that *Ggnbp2* resection in mice caused dramatic structural defects during spermatid metamorphosis and exhibited a complete absence of mature spermatozoa phenotype.^4^ Given the evidence that GGNBP2 immunoreactivity was strongest in pachytene spermatocytes^4^, ^13^ and the number of spermatids started to decline at the initial stage despite *Ggnbp2* null mutants having a complete continuous process of spermatogenesis,^4^ which raises the possibility that *Ggnbp2* loss may compromise spermatocyte meiosis during spermatogenesis.

Meiotic homologous recombination of spermatocytes is important for proper chromosomal segregation during spermatogenesis to generate haploid spermatids and is highly regulated to ensure at least one crossover per homolog pair. Meiotic division is induced by programmed DNA double‐strand breaks (DSB) that form at preferred sites or hotspots.^15^, ^16^, ^17^ A small number of DSBs form crossover recombinations, and the majority of DSBs are repaired as non‐crossover recombinations in spermatocytes.^17^, ^18^ These processes involve a number of meiotic‐specific and ubiquitously expressed nuclear proteins, such as γH2AX, RAD51, RAD18, DMC1, BRCA1 and MLH1.^19^, ^20^, ^21^, ^22^ These proteins are co‐ordinately recruited to DSB sites to facilitate DNA DSB repair via homologous recombination. Defects in DNA DSB repair can cause chromosome mis‐segregation underling aneuploidy‐related pathologies or meiotic arrest of spermatocytes leading to male infertility.^17^, ^20^, ^23^, ^24^

Gametogenetin (GGN) is a testis‐enriched germ cell‐specific protein and has three splice variants.^25^ The largest variant GGN1 is demonstrated to be the predominant form in the testis and localizes in spermatocytes, spermatids and spermatozoa but not in spermatogonia in mouse and human testes.^25^, ^26^, ^27^ GGN1 is identified to interact with various proteins, which include FANCL, FANCD2, BRCC36, OAZ3, CRISP2, GGNBP1 and GGNBP2.^26^, ^28^, ^29^, ^30^ As such, GGN1 is proposed to play a role in meiosis involving in DNA DSB repair^25^, ^27^, ^30^ as well as sperm tail development and/or motility in the testis.^29^ Complete deletion of *Ggn* leads to embryonic lethality before implantation. Haploinsufficiency of *Ggn* in mouse pachytene spermatocytes results in an increased incidence of unrepaired DSBs, which supports that GGN1 plays a role in spermatogenesis, especially in DNA DSB repair during spermatocyte meiosis.^30^

This study aims to determine whether meiotic progression of spermatocytes is compromised in *Ggnbp2KO* mice and provides further biological insight into the function of GGNBP2 in spermatogenesis. Here, we report that in addition to its indispensability in spermatid transformation, GGNBP2 plays a role in DNA DSB repair during spermatocyte meiosis, which is likely mediated by interaction with GGN1 to modulate its nuclear translocation and consequently affect the DNA DSB repair activity.

## 2 MATERIALS AND METHODS

### 2.1 Mice and cell line

The *Ggnbp2* Knockout (*Ggnbp2KO*) mice with a mixed C57BL/6/129/Sv genetic background were generated as previously described.^4^ Mouse tail genomic DNA was extracted using ZR genomic DNA‐tissue mini prep kits according to the procedure recommended by the manufacturer (Zymo Research Corp, Irvine, CA, USA) and genotyped by PCR using primer sets as listed in Table 1. Mice were housed on a 12‐hour light/12‐hour dark cycle with food and water provided ad libitum. The studies have been approved by the Animal Care and Use Committee of the University of Louisville. All mice were killed under ketamine anaesthesia and efforts were taken to minimize their discomfort.

**Table 1:** Primer sequences for genotyping and RT‐PCR

Gene	Primer sequence	PCR cycles	Size (bp)
*Ggnbp2 (genotyping)*	F: 5′ AGTGCCATTTACCCACCAAG 3′ R: 5′ GAAAGGAGGAGGGAAAGGAA 3′	30	417
*Ggnbp2 (RT‐PCR)*	F: 5′ CCTGACGATGGTGATGGAATTT 3′ R: 5′ CTCCCTTGGGCCCTACT 3′	30	275
*Acrv1 (RT‐PCR)*	F: 5′ TTCTCAGCTCTTGAGTGTGC 3′ R: 5′ TGTGTTCTGAAGACTGTTCTCC 3′	30	344
*Prm2 (RT‐PCR)*	F: 5′ ATGGTTCGCTACCGAATGAGG 3′ R: 5′ ATGATGTTGCTTGGGCAGGT 3′	31	405
*Pms2 (RT‐PCR)*	F: 5′ TAATCAGCTCGGACAGGGGA 3′ R: 5′ ACGGAAACGTTAAGGACGACA 3′	31	372
*Ggn (RT‐PCR)*	F: 5′GGCTGAGATAATATTGGAGGACAG 3′ R: 5′ GAAGAGCCCGTGGGTTT 3′	30	500
*Pem (RT‐PCR)*	F: 5′ TGCTCCTGGTCCTATGGGTGATG 3′ R: 5′ CCGCAGCCCTCCTGATCTTAAAC 3′	32	352
β*‐Actin (RT‐PCR)*	F: 5′ GCTGTCCCTGTATGCCTCTGGT 3′ R: 5′ CACGCACGATTTCCCTCTCA 3′	30	215
*Rpl19 (RT‐PCR)*	F: 5′ CTCAGGCTACAGAAGAGGCTT 3′ R: 5′ GGACAGAGTCTTGATGATCTC 3′	30	560

F, forward; R, reverse.

Immortalized murine spermatocyte cell line GC‐2spd cells were procured from American tissue culture collection (Manassas, VA, USA). GC‐2spd cells were maintained in Dulbecco's modified Eagle's medium (DMEM, Sigma, St Louis, MO, USA) supplemented with 10% heat‐inactivated foetal bovine serum (Sigma) and 1% penicillin/streptomycin (Invitrogen, San Francisco, CA, USA) at 37°C (unless indicated elsewhere) in a humidified incubator containing 5% CO_2_ and 95% atmospheric air.

### 2.2 Stable transfection of *Ggnbp2* and *Ggn* CRISPR knockout plasmids

*Ggnbp2* (sc‐432161‐NIC) and *Ggn* double nickase plasmids (sc‐434015‐NIC) were purchased from Santa Cruz Biotech (Dallas, TX, USA). The day before *Ggnbp2* or *Ggn* plasmid transfection, GC‐2spd cells were seeded into a 6‐well plate at a density of 2 × 10^5^ per well. Prior to transfection, the growth medium was replaced with fresh antibiotic‐free growth media (Sigma). 300 μL plasmid DNA/UltraCruz transfection reagent complexes (Santa Cruz Biotech) were added to each well, followed by incubation overnight. The medium was replaced by the growth medium 24 hours post‐transfection. Three days after transfection, the medium was replaced with fresh medium containing 4 μg puromycin/mL (Sigma). After 2 weeks of puromycin selection, green fluorescent protein (GFP) positive and puromycin‐resistant clones were dispersed by trypsin digestion (Sigma) and transferred to 96‐well plates at a density of 1‐2 cells per well. The procedure of single cell cloning was repeated three times to obtain 100% clonal purity. The stable *Ggnbp2 and Ggn* deleted cell lines (*Ggnbp2* ^*−/−*^ and *Ggn* ^*−/−*^) were verified by RT‐PCR and Western blotting. GC‐2spd cells transfected with a control double nickase plasmid that contains a non‐targeting scramble guide RNA was used for a control.

### 2.3 Propidium iodide staining and flow cytometric assay

For flow cytometric analysis, testicular germ cell suspension was prepared. Briefly, the decapsulated testes were incubated in phosphate‐buffered saline (PBS) containing 0.5 mg/mL collagenase (type IV) for 15 minutes followed by digestion with DNase I (1.0 μg/mL) and trypsin (1.0 μg/mL) in PBS for an additional 15 minutes. The incubations were carried out in a shaking water bath at 32°C. The suspension was then filtered through 80 μm nylon meshes. After washing twice with PBS, the germ cells were fixed in 70% ice‐cold ethanol overnight at 4°C. For GC‐2spd cell flow cytometry, cells were seeded in 6‐well plates at a density of 2 × 10^4^ cells/cm^2^ with 3 mL DMEM medium per well and cultured at 32°C. When cell growth reached 80%‐90% confluence, the cells were harvested and fixed in 70% ice‐cold ethanol overnight at 4°C. The fixed germ and GC‐2spd cells were washed twice with ice‐cold PBS and stained with 50 mg/mL propidium iodide (PI, Sigma) containing 50 mg/mL RNase A (Sigma) for 30 minutes in the dark. Flow cytometric assays were performed with a FACS Vantage flow cytometer (BD Bioscience, San Jose, CA, USA) and analysed using FlowJo software (TreeStar, Ashland, OR, USA). The experiments were repeated three times for each of three samples of wild‐type (WT) and *Ggnbp2KO* testicular germ cells, *Ggnbp2* ^*+/+*^ and *Ggnbp2* ^*−/−*^ GC‐2spd cells colonies and *Ggn* ^*+/+*^ and *Ggn* ^*−/−*^ GC‐2spd cells colonies.

### 2.4 RT‐PCR

Total RNA was extracted from the testes using TRIzol reagent (Invitrogen) according to the manufacturer's instructions. Two microgram total RNA was reverse transcribed to cDNA with random primers (Invitrogen) and avian myeloblastosis virus reverse transcriptase (MP Biochemicals, Santa Ana, CA, USA). The cDNA was amplified by PCR with the primer sets of the target gene and housekeeping genes. PCR cycle consisted of denaturation for 45 seconds at 94°C, annealing for 1 minutes at 57°C and extension for 1 minutes 30 seconds at 72°C. The amplified products were separated by electrophoresis in 1% agarose gels and stained by ethidium bromide. The intensity of specific bands was scanned and semiquantified using the image analysis software Total Lab (Nonlinear USA Inc., Durham, NC, USA). The results were presented as the ratio of the target gene over a housekeeping gene, β‐actin or *Rpl19*. All RT‐PCR primers, as listed in Table 1, were designed according to the sequences obtained from GenBank and synthesized by Operon Technologies (Alameda, CA, USA).

### 2.5 Preparation of testicular germ cells and nuclear spread slides and immunofluorescence staining

Dispersed testicular germ cells were prepared by incubation of the seminiferous tubules in PBS supplemented with 0.5% bovine serum albumin, 2 mg/mL collagenase, 0.1 μg/mL DNase I and 0.5% trypsin (Sigma) for 10 minutes at 32°C. The dissociated cells were then resuspended in 4% paraformaldehyde, washed with PBS, spread on slides and air‐dried.

Nuclear spreads were prepared by the “drying down technique” as previously described.^31^ Briefly, the seminiferous tubules were isolated from 28‐day‐old testes. The germ cells were dissociated from the seminiferous tubules by repeatedly up‐down pipetting in PBS. The cells were ruptured by incubation with a hypotonic buffer (30 mmol/L Tris, 50 mmol/L sucrose, 17 mmol/L trisodium citrate dihydrate, 5 mmol/L Ethylenediaminetetraacetic acid (EDTA), 0.5 mmol/L Dithiothreitol and 0.5 mmol/L phenylmethylsulfonyl fluoride, pH 8.2) for 30 minutes at room temperature (RT) and then fixed with 4% paraformaldehyde for 30 minutes before spreading on the slides. The slides were incubated in a humidified chamber for 1 hour and then dried for 30 minutes at RT. Finally, the slides were washed twice in 0.4% Photoflo (Kodak) and air‐dried.

SYCP3 was detected by a monoclonal antibody and labelled by tetramethylrhodamine (TRITC)‐conjugated donkey anti‐mouse IgG (red colour). The other proteins were detected by polyclonal antibodies and stained by fluorescein isothiocyanate (FITC)‐conjugated bovine anti‐rabbit IgG (green colour). The primary antibodies used in our experiment are listed in Table 2. FITC‐conjugated peanut agglutinin (PNA)‐lectin (green colour) was used to label acrosomes (Molecular Probes, Eugene, OR, USA). All slides were covered with a 4′,6‐diamidino‐2‐phenylindole (DAPI) containing mounting medium (Santa Cruz Biotech) to visualize the nuclei (blue colour). Fluorescent signals were visualized and photographed using an Olympus fluorescence microscope (IX71/DP72, Tokyo, Japan). Analyses of images were performed with ImageJ version 1.51p. Replacement of the primary antibody with irrelevant mouse or rabbit IgG was used as a procedure control.

**Table 2:** Antibodies used in this report

Antibody	Host species	Vendor	Catalog NO.	Application	Dilution
GGNBP2	Rabbit	Pacific Immuno. Corp.	PAC‐139	WB IP	1:3000 1 μg/0.2 mg protein
SYCP3	Mouse	Santa Cruz Biotech.	SC‐74569	IF	1:400
SYCP3	Rabbit	GeneTex	GTX15092	IF	1:400
SYCP1	Rabbit	GeneTex	GTX15087	IF	1:200
GGN	Rabbit	Aviva System Biology	OABF00642	IF IP	1:200 1 μg/0.2 mg protein
GGN	Rabbit	Aviva System Biology	ARP53093_P050	WB	1:3000
GAPDH	Mouse	Santa Cruz Biotech.	SC‐47724	WB	1:3000
His‐tag	Mouse	Aviva System Biology	OAEA00010	WB	1:1500
FANCL	Mouse	Santa Cruz Biotech.	SC‐66887	WB IP	1:600 1 μg/0.2 mg protein
β‐Actin	Mouse	Santa Cruz Biotech.	SC‐130300	WB	1:3000
RAD18	Rabbit	ABclonal	A5380	IF IP	1:200
RAD51	Rabbit	Millipore	ABE257	IF	1:200
RAD51	Mouse	Santa Cruz Biotech.	Sc‐398287	IP	1 μg/0.2 mg protein
γ‐H2AX	Rabbit	BETHY	A300‐081A‐T	IF	1:3000

IF, immunofluorescence staining; IP, immunoprecipitation; WB, Western blot.

### 2.6 Western blotting

Testes were homogenated using a Tissue‐Tearor (RPI Corp, Mt. Prospect, IL, USA) in lysis buffer containing 50 mmol/L Tris‐HCl, 150 mmol/L NaCl, 1% NP‐40, 1 mmol/L EDTA, 2 mmol/L MgCl_2_, 0.5% sodium deoxycholate, complete protease inhibitor and phosphorylase inhibitor cocktail (Roche, Mannheim, Germany). The protein concentrations were determined using Bradford protein assay kits (Bio‐Rad, Hercules, CA, USA). 20 μg testicular homogenates each sample was electrophoresed in 10% SDS/PAGE gels and then electroblotted to TransBlot membranes (Bio‐Rad). After blocking with 3% non‐fat milk in Tris‐buffered Saline‐Tween‐20, membranes were incubated with the primary antibody as listed in Table 2, followed by horseradish peroxidase (HRP)‐conjugated secondary antibodies (Santa Cruz Biotech). Signal was detected using a Pierce ECL Western blotting substrate detection kit (Thermo Fisher Scientific, Waltham, MA, USA). All membranes were reblotted with β‐actin or glyceraldehyde 3‐phosphate dehydrogenase (GAPDH) antibody as the loading control. The intensity of specific bands was scanned using image analysis software Total Lab. The results were presented as the ratio of target protein over β‐actin or GAPDH.

### 2.7 Immunoprecipitation

Equal amount of testicular lysate (200 μg) was added to 20 μL protein A/G Plus‐agarose beads (Santa Cruz Biotech) for 1 hour at 4°C with constant agitation on a rotator to remove the non‐specific interacting proteins. Following a brief centrifugation, the supernatant was then incubated with 1 μg of anti‐GGN or anti‐GGNBP2 antibody for 4 hours at 4°C with constant agitation. Thereafter, 20 μL of protein A/G Plus‐agarose beads were added to each sample and incubated at 4°C with constant agitation overnight. The samples were then washed subsequently with buffer A (50 mmol/L Tris‐HCl, 0.5% Triton X‐100, 1 mmol/L EDTA, 150 mmol/L NaCl, pH 8.0) and buffer B (10 mmol/L Tris‐HCl, 0.1% Triton X‐100, pH 7.5). Normal rabbit IgG was used as a negative control. Bound protein‐agarose complexes were boiled and separated on SDS‐PAGE gels. Immunoprecipitated proteins were electroblotted to TransBlot membranes and immunoblotted with the corresponding antibodies as listed in Table 2.

### 2.8 Transient transfection of *Ggnbp2* expression plasmids

The *Ggnbp2* cDNA fragment was obtained by PCR using testis cDNA as the template and validated by DNA sequencing. The correct full‐length *Ggnbp2* cDNA was cloned into *pcDNA3–6HisC* vector (Invitrogen) to construct the mammalian expression plasmid *pcDNA3–HisC tagged‐Ggnbp2* (*pcDNA3–Ggnbp2*). *Ggnbp2* ^*−/−*^ or *Ggn* ^*−/−*^ GC‐2spd cells were cultured in DMEM medium to approximately 70% confluence in a 6‐well plate and were transfected with 2 μg of either *pcDNA3–Ggnbp2* or *pcDNA3* plasmid (as a control) with a plasmid transfection reagent (Santa Cruz Biotech). The cells were cultured in the same medium for 72 hours, then harvested for Western blot and flow cytometric assays.

### 2.9 Statistics

All statistical analyses were performed with a version 3.06 Instat program (GraphPad Software, San Diego, CA, USA). Comparisons of means between more than two groups were performed by one‐way analysis of variance (ANOVA). Student's *t* test was used when comparing means of two groups. A *P*‐value <.05 was considered statistically significant.

## 3 RESULTS

### 3.1 *Ggnbp2KO* leads to aberrant distribution of testicular germ cells

**Figure 1:**
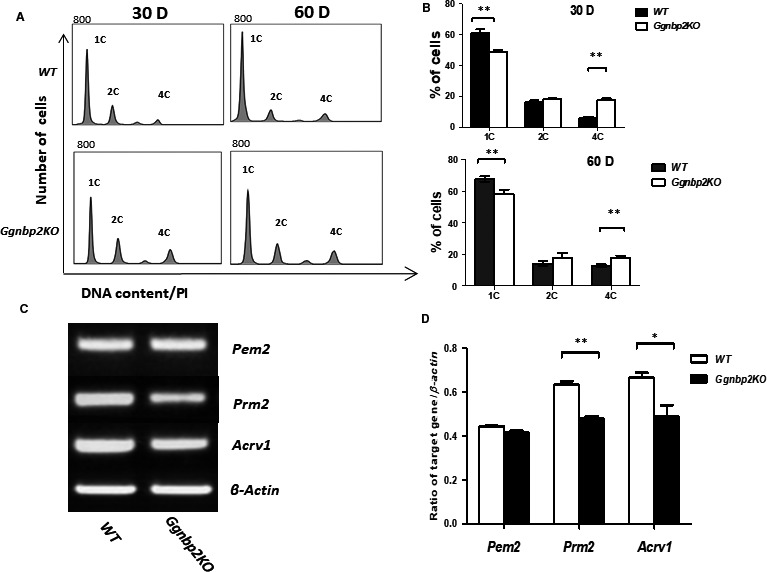
Lack of *Ggnbp2* leads to aberrant distribution of testicular germ cells. A,B, Representative histograms of flow cytometric analyses of the DNA contents of propidium iodide (PI)‐stained testicular germ cells isolated from 30‐ (30 D) and 60‐d (60 D)‐old wild‐type (WT) and *Ggnbp2* knockout (*Ggnbp2KO*) mice (A). Cell populations with different DNA contents are denoted as haploid (1C), diploid (2C) and tetraploid peaks (4C). Quantification of three biological replicates shows a significant decrease in haploid cells, while a significant increase in tetraploid cells in *Ggnbp2KO* compared to WT mice (B). C,D, Representative RT‐PCR picture (C) and β‐actin normalized data (D) of three 60‐d‐old mice demonstrate a decrease in mRNA levels of spermatid marker genes *Prm2* and *Acrv1*, while a Sertoli cell marker gene *Pem2* mRNA level is not significantly different between *Ggnbp2KO* and WT mice. Data are presented as mean ± SEM. (**P < .01, *P < .05)

We previously reported that *Ggnbp2KO* resulted in an absence of mature spermatozoa and a significant reduction in spermatids in the adult testes using morphometric analysis methods.^4^ In this study, we examined the DNA contents of testicular germ cells isolated from 30‐ and 60‐day‐old WT and *Ggnbp2KO* mice using flow cytometry. *Ggnbp2KO* mice showed markedly decreased haploid (1C) cell fraction and increased tetraploid (4C) cell fraction compared to age‐matched either 30‐ or 60‐day‐old WT littermates (Figure 1A,B). In agreement with this data, RT‐PCR analysis of 60‐day‐old *Ggnbp2KO* testes showed a significant decrease in both round and elongated spermatid markers *Prm2* and *Acrv1* (Figure 1C,D).

### 3.2 *Ggnbp2KO* does not impact chromosome synapsis, but results in compromised meiotic DSB repair

**Figure 2:**
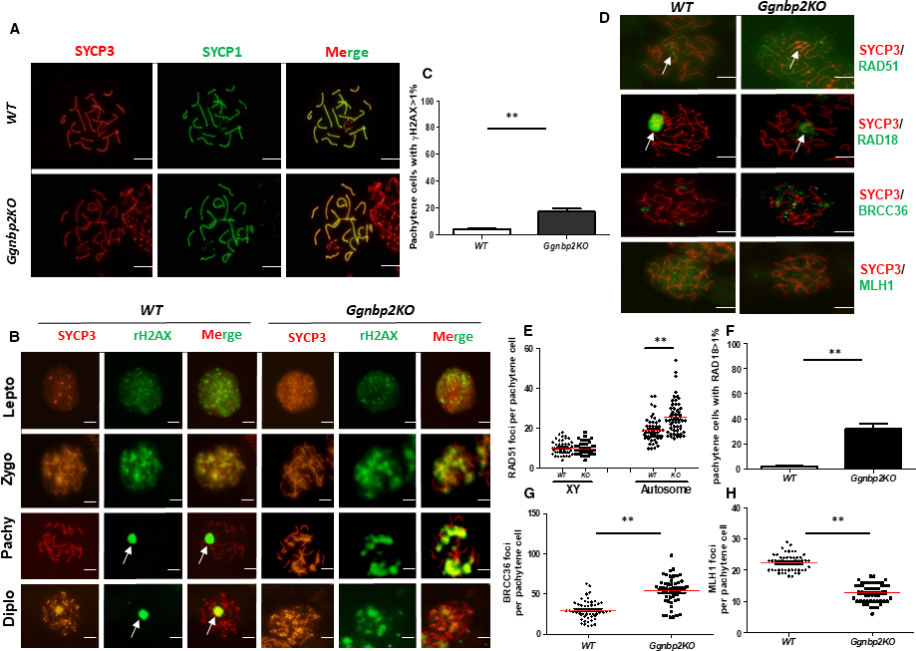
*Ggnbp2* deletion does not affect synapsis but compromises the repair of meiotic DNA double‐strand breaks (DSB). A, Nuclear spreads of spermatocytes stained with antibodies against SYCP1 (red) and SYCP3 (green) show that the assembly and disassembly of the synaptonemal complexes are not affected. B, Chromosome spreads of spermatocytes are stained with antibodies against SYCP3 (red) and γH2AX (green). Four meiotic stages of spermatocyte, leptotene (lepto), zygotene (zygo), pachytene (pachy) and diplotene (diplo) are presented. D, Chromosome spreads of pachytene spermatocytes are stained with SYCP3 antibody (red), DNA DSB repair protein antibodies RAD51, RAD18 and BRCC36 and a crossover marker MLH1 (green) as indicated. Arrows indicate the XY body stained by γH2AX, RAD51 and RAD18. C,F, The percentage of pachytene cells with >1 γH2AX (C) and >1 RAD18 foci (F) are significantly higher in *Ggnbp2KO* than WT mice, which is calculated from 100 randomly selected pachytene spermatocytes per testis and data are from three 60‐d‐old testes. E,G,H, The numbers of RAD51 (E), BRCC36 (G) and MLH1 (H) foci counts per pachytene cell are significantly higher in *Ggnbp2KO* than WT mice, which is counted from 50 randomly selected pachytene spermatocytes per testis and results are from three 60‐d‐old testes. Data are presented as the mean ± SEM (**P < .01, scale bar = 5 μm)

To determine whether deletion of *Ggnbp2* causes meiotic defects, we examined the dynamics of spermatocyte chromosome synapsis and recombination using antibodies directed against the synaptonemal complex proteins, axial/lateral element SYCP3 and central element SYCP1, which are main components of the meiotic nodules and chromatin markers.^32^ The labelling pattern of SYCP1 and SYCP3 was colocalized and the mutant spermatocytes were indistinguishable from that of WT littermates (Figure 2A), suggesting that *Ggnbp2* loss does not affect the formation and disassembly of the synaptonemal complexes.

We next examined whether *Ggnbp2KO* affected meiotic DSB repair. γH2AX is a meiotic marker that rapidly appears in the form of nuclear foci concomitantly with DNA DSB formation and repair.^33^, ^34^ In WT spermatocytes, globally distributed γH2AX foci were observed during the leptotene, correlating with DNA DSB formation, then diminished as breaks were repaired during the zygotene, eventually concentrated in the bivalent XY body by the pachytene and diplotene stages (Figure 2B). Although staining pattern of γH2AX in *Ggnbp2KO* spermatocytes in the leptotene stage was similar to that of WT, its staining persisted not only in the XY body but also in several patches along autosomes in the pachytene and diplotene stages (Figure 2B). Pachytene cells that contain more than one γH2AX foci in *Ggnbp2KO* spermatocytes were significantly higher than that of WT (Figure 2C). In addition, we checked the distribution of several DNA DSB repair and meiotic recombination marker molecules RAD51, RAD18, BRCC36 and MLH1. The immunostaining pattern showed that RAD51 foci per pachytene cell on the XY body were not significantly different between the two groups. In contrast, a marked increase in autosomal RAD51 foci was observed in the *Ggnbp2KO* spermatocytes (Figure 2D,E). In WT spermatocytes, RAD18 was accumulated as RAD51‐like foci on the synaptonemal complexes only seen in the zygotene stage and was restricted to the XY body in the pachytene stage (Figure 2D). In *Ggnbp2KO* pachytene spermatocyte, RAD18 staining was sustained in synapsed autosomal homologs in addition to the XY body (Figure 2D,F). BRCC36 is a major component of the BRCA1‐A complex, which participates in promoting BRCA1 function in response to DNA damage.^35^ Immunofluorescence staining revealed that BRCC36 foci dramatically increased (Figure 2D,G) and MLH1 foci, a marker of chiasma formation, significantly decreased in *Ggnbp2* null pachytene cells (Figure 2D,H).

### 3.3 GGNBP2 interacts with GGN1 and both have the same subcellular localization during spermatogenesis

**Figure 3:**
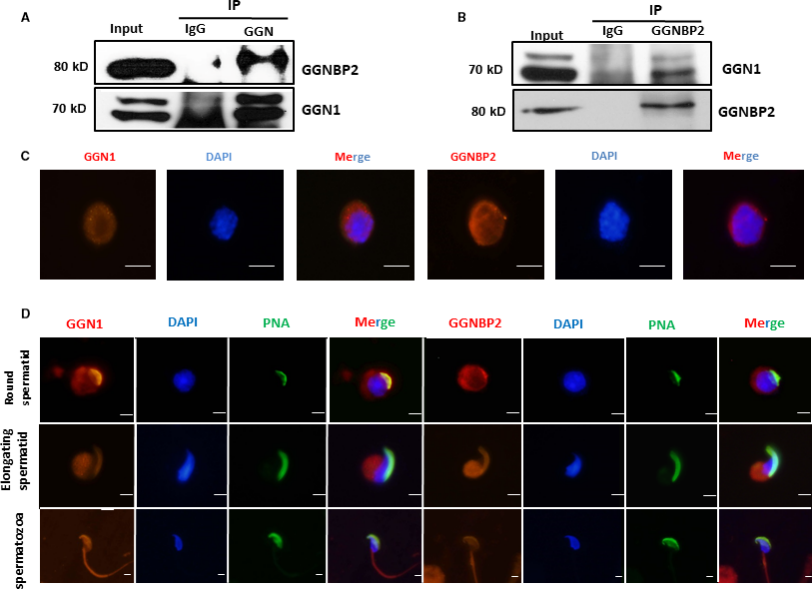
Co‐immunoprecipitation of GGNBP2 and GGN1 in the mouse testis and localization of GGNBP2 and GGN1 in spermatogenic cells. A,B, Interaction of GGNBP2 and GGN1 in the mouse testis is determined by immunoprecipitation (IP) with GGN1 (A) or GGNBP2 (B) antibody, and cell lysates (input) and immunoprecipitated protein complexes are immunoblotted as indicated. C, Both GGN1 and GGNBP2 are localized in the nuclei and cytoplasm of spermatocytes. D, Both GGN1 and GGNBP2 localization in round spermatid, elongating spermatid and spermatozoa. PNA lectin (green) is used to label acrosomes, and DAPI is used to label the nuclei (blue). Representative of three experimental replicates. Scale bar = 5 μm

It has been reported that GGNBP2 interacts with testicular proteins, GGN1, GGNBP1, CRISP2 and OAZ3.^26^, ^28^, ^29^, ^30^ Reciprocal immunoprecipitation (IP) with GGN (Figure 3A) and GGNBP2 (Figure 3B) antibodies demonstrated that GGN1 and GGNBP2 formed a protein complex in the testis. Our previous use of immunohistochemical staining showed that GGNBP2 predominately localized in spermatocytes and round spermatids,^4^ and GGN1 was reported to detect in spermatocytes, spermatids and sperm.^28^ To determine subcellular localizations of GGNBP2 and GGN1 in spermatocytes, spermatids and spermatozoa, dispersed testicular cells were stained by immunofluorescence. Both GGNBP2 and GGN1 were observed in the nucleus and cytoplasm of spermatocyte and they colocalized very well with each other (Figure 3C). In round and elongating spermatids, both GGNBP2 and GGN1 were detected in the nucleus and cytoplasm as well as acrosome. Immunofluorescence signal of these two proteins was found in the tail of spermatozoa in addition to the nucleus and acrosome (Figure 3D). These results suggest GGNBP2 shares similar trafficking dynamics with GGN1 during spermatogenesis.

### 3.4 *Ggnbp2KO* results in a decrease in *Ggn* mRNA and protein levels

**Figure 4:**
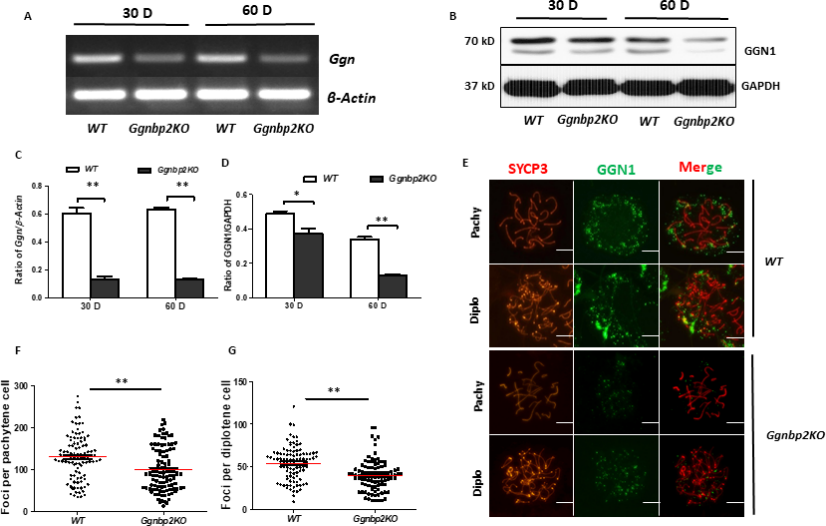
*Ggnbp2* null mutation affects *Ggn* mRNA and protein levels and nuclear accumulation of GGN1. A‐D, RT‐PCR and Western blot demonstrate a decrease in *Ggn* mRNA (A,C) and protein (B,D) levels in the testes of 30‐d (30 D) and 60‐d (60 D)‐old *Ggnbp2KO* mice. β‐actin normalized *Ggn* mRNA levels (C) and GAPDH‐normalized GGN1 protein (D) levels are from three biological replicates. E, Spermatocytes of 60‐d‐old WT and *Ggnbp2KO* stained with antibodies against SYCP3 (red), and GGN1 (green) in three meiotic stages, pachytene (pachy) and diplotene (diplo) show commensurately fewer GGN1 are recruited onto the chromosomes in *Ggnbp2KO* spermatocytes. F,G, GGN1 foci numbers per pachytene (F) and per diplotene (G) cells are counted from 100 randomly selected spermatocytes per testis, and results are from three mice. Data are presented as the mean ± SEM (**P < .01, *P < .05, scale bar = 5 μm)

To assess whether *Ggn* expression was affected due to *Ggnbp2* loss, RT‐PCR and Western blots were performed in 30‐ and 60‐day‐old testes. Both mRNA (Figure 4A,C) and protein (Figure 4B,C) levels in the *Ggnbp2KO* testes were noticeably reduced compared to WT testes. Immunofluorescence staining of GGN1 in nuclear spread testicular germ cells revealed that GGN1 immunostaining localized in the cytoplasm surrounding the nucleus and GGN1 foci were present in the nucleus associated with chromosomes in WT spermatocytes (Figure 4E). Overall immunofluorescence intensity of GGN1 in *Ggnbp2KO* spermatocytes was remarkably reduced (Figure 4E). GGN1 foci that associated with chromosomes in the nucleus were significantly lower than that of WT spermatocytes (Figure 4E‐G).

### 3.5 *Ggnbp2* and *Ggn* knockout share the similar phenotype in GC‐2spd cells

**Figure 5:**
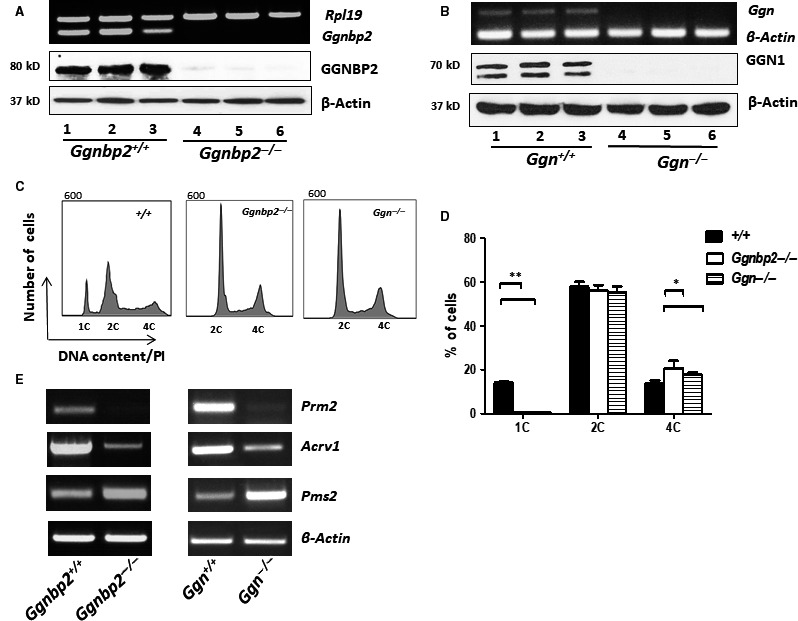
Either *Ggnbp2* or *Ggn* inactivation inhibits meiotic differentiation of GC‐2spd cells. A,B, Three representative stable GC‐2spd cell clones transfected with *Ggnbp2* (*Ggnbp2* ^*−/−*^) or *Ggn* (*Ggn* ^*−/−*^) CRISPR double nickase knockout plasmids contain no detectable *Ggnbp2* (A) or *Ggn* (B) transcripts and proteins demonstrated by RT‐PCR and Western blot, respectively. C,D, Flow cytometric results reveal that significant reduction in haploid cell number in the absence of either *Ggnbp2* or *Ggn* in GC‐2spd cells. Representative histograms (C) and quantitative data (D) are from three experimental replicates. E, RT‐PCR analyses show a remarkable decrease in mRNA levels of spermatid marker genes *Prm2* and *Acrv1*, while spermatocyte marker gene *Pms2* mRNA levels are increased in both *Ggnbp2* ^*−/−*^ and *Ggn* ^*−/−*^ GC‐2spd cells. The experiments are repeated three times and data are presented as mean ± SEM. (**P < .01, *P < .05)

**Figure 6:**
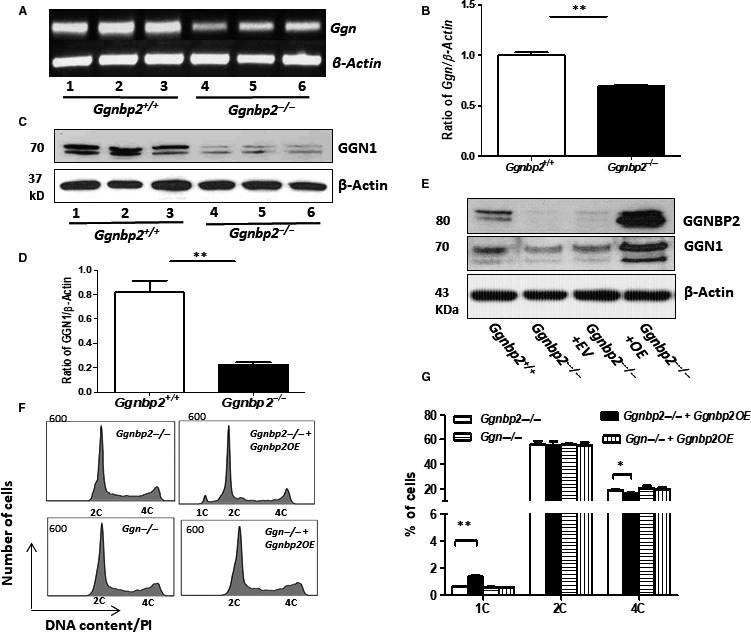
Enforced expression of *Ggnbp2* in *Ggnbp2* ^*−/−*^ GC‐2spd cells restores *Ggn* expression and increases haploid differentiation. A‐D, *Ggnbp2* deletion decreases *Ggn* mRNA (A,B) and protein expression (C,D) in three stable *Ggnbp2* ^*−/−*^ GC‐2spd cell clones. E, *Ggnbp2* ^*−/−*^ GC‐2spd cells are transfected with *pCDNA3‐HisC* empty vectors (EV) or *pCDNA3‐His‐Ggnbp2* expression plasmids (OE). *Ggnbp2* overexpression increases *Ggn* protein determined by Western blot. F,G, Representative histograms (F) and quantitation (G) of flow cytometric assays show *Ggnbp2* overexpression increases the number of haploid cells in *Ggnbp2* ^*−/−*^ but not in *Ggn* ^*−/−*^ GC‐2spd cells. The experiments are repeated three times, and data are presented as mean ± SEM. (**P < .01, *P < .05)

Immortalized GC‐2spd cell line is originally derived from mouse spermatocytes.^36^ It has been shown that these cells are capable of undergoing meiosis to produce a haploid population, expressing some post‐meiotic marker genes and possessing morphological features in common with early spermatids following low‐temperature culture (32°C).^37^ To evaluate the role of GGNBP2 and GGN1 in spermatocyte meiotic differentiation, we generated stable *Ggnbp2* and *Ggn* knockout (*Ggnbp2* ^*−/−*^ and *Ggn* ^*−/−*^) GC‐2spd cells using CRISPR/Cas9 technique, respectively. Complete abolishment of *Ggnbp2* and *Ggn* expression was verified by RT‐PCR and Western blot (Figure 5A,B). Flow cytometric analysis of the DNA contents showed that either *Ggnbp2* or *Ggn* deletion resulted in a significant reduction in haploid cells (1C) compared to *Ggnbp2* ^*+/+*^ and *Ggn* ^*+/+*^ counterparts, while the tetraploid (4C) cells increased and diploid cells (2C) were not significantly changed (Figure 5C,D). Both of the haploid spermatid marker genes, *Prm2* and *Acrv1*, were noticeably down‐regulated, while the spermatocyte marker gene *Pms2* was increased in both *Ggnbp2* ^*−/−*^ and *Ggn* ^*−/−*^ GC‐2spd cells. The same was observed in the testis, *Ggnbp2* loss in GC‐2spd cells also impeded *Ggn* expression (Figure 6A‐D).

We next asked whether re‐expression of *Ggnbp2* would rescue the phenotype of *Ggnbp2* ^*−/−*^ and *Ggn* ^*−/−*^ GC‐2spd cells. The results showed that GGNBP2 protein was readily detected in *Ggnbp2* ^*−/−*^ GC‐2spd cells following transfection of *Ggnbp2* expression plasmids (Figure 6E). Interestingly, an increased GGN1 protein level was observed in overexpressed *Ggnbp2* ^*−/−*^ GC‐2spd cells (Figure 6E). More importantly, enforced expression of *Ggnbp2* in *Ggnbp2* ^*−/−*^ GC‐2spd cells restored these cells undergoing meiosis to produce a haploid population (Figure 6F,G). However, enforced expression of *Ggnbp2* in *Ggn* ^*−/−*^ GC‐2spd cells did not improve meiotic arrest phenotype.

## 4 DISCUSSION

In this report, we explored the role of GGNBP2 in meiotic progression of spermatocytes. In our previous study, based on staging analyses of cross sections of the seminiferous tubules, the numbers of spermatogonia, spermatocytes and Sertoli cells were not significantly different between *Ggnbp2KO* and WT mice. In contrast, a marked decline of spermatids in each stage of the seminiferous tubules was observed. In the present study, we use flow cytometry to analyse the DNA contents of germ cells of two groups and found that the amount of diploid (2C) cells that may include spermatogonia, secondary spermatocytes and Sertoli cells, is not different between WT and knockout mice. This is consistent with previous histological observation of a defect in spermatid differentiation.^4^ In contrast, the amount of tetraploid (4C) cells is significantly greater in *Ggnbp2* null testes than that of WT littermates. In agreement with the in vivo data, our in vitro results also demonstrated that *Ggnbp2* knockout in GC‐2spd cells inhibited spermatocyte differentiation into haploid (1C) cells that led to an increase in tetraploid (4C) cells. A decrease in haploid cells accompanied by an increase in tetraploid cells in the *Ggnbp2* null testes suggests that GGNBP2 may affect the prophase I of male meiosis in addition to spermatid differentiation.

Meiosis in prophase I involves a series of orchestrated and programmed biochemical events including generation of DNA DSBs, synapsing of the homologous chromosomes, meiotic sex chromosome inactivation and repair of DNA DSBs.^15^, ^16^ Meiotic recombination starts with the programmed formation of a DNA DSB, which is catalysed by the meiotic topoisomerase‐like protein SPO11.^38^, ^39^, ^40^ Concomitantly, a number of DNA damage response factors, including RAD51 and BRCC36, associate with chromatin enriched in γH2AX. In our *Ggnbp2KO* model, induction of DNA DSBs appears to be generated on time, which is manifested by accumulation of γH2AX at leptotene and zygotene stages.^19^ Synapsis and meiotic sex chromosome inactivation seem to occur normally. However, inappropriately accentuated and persistent autosomal γH2AX staining in pachynema and even diplonema point to DNA DSB repair defect as one primary cause of meiotic impairment in the absence of GGNBP2. This is further buttressed by the evidence that commensurately increases in cytological markers for DSB repair proteins RAD51, RAD18 and BRCC36 in meiocytes. BRCC36 is a major component of the BRCA1‐A complex, which participates in promoting BRCA1 function in response to DNA damage.^35^ The RAD18 is expressed highest levels in the testis.^41^ Its recruitment to meiotic DSBs is thought to occur if these breaks persist.^21^ Hence, a decrease in MLH1 focus formation may possibly be caused by the inefficient DSB repair that likely promotes an aberrant activity of alternative DNA repair via the non‐homologous end joining pathway^17^ and results in a reduction in crossovers in the absence of GGNBP2.

GGN1 is known to contain nuclear targeting signalling and GGNBP2 has a zinc finger motif in the N‐terminus and a nuclear protein‐binding domain in the C‐terminus.^3^, ^9^, ^25^ Current and previous studies demonstrate that GGNBP2 and GGN1 have a similar testicular expression profile and interact specifically in the testis. The highest levels of these two proteins are found in meiotic spermatocytes and persisted through spermatids and displayed a developmentally translocation between the nucleus and cytoplasm during meiosis and spermiogenesis. *Ggnbp2* loss profoundly reduces GGN1 nuclear localization suggesting that binding of GGNBP2 to GGN1 may be necessary for nuclear accumulation. There is the possibility that GGNBP2 regulates the distribution, thus the activity of GGN1. Intriguingly, *Ggn* expression is down‐regulated in *Ggnbp2* null testes implying that GGN1‐GGNBP2 complex may be also required for *Ggn* expression. More importantly, *Ggn* resection in GC‐2spd cells essentially replicates the *Ggnbp2* deletion phenotype, while enforced expression of *Ggnbp2* in *Ggnbp2* null GC‐2spd cells but not in *Ggn* null GC‐2spd cells partially rescues the defect in differentiation of spermatocytes into haploid cells. The intimate expression and physical relation between GGNBP2 and GGN1 and loss of either protein in GC‐2spd cells displaying similar phenotypes indicate that the role of GGNBP2 in DNA DSB repair during spermatocyte meiosis appears to be mediated through GGN1. GGNBP2 and GGN1 may work consonantly in cell traffic. GGN1, in turn, recruits other factors to proper positions on the chromosome to repair DSBs. Jamsai et al^30^ proposed that GGN1 played a role in meiotic DSB repair in the testis via its connection with the factors such as FANCL and BRCC36 of Fanconi anaemia (FA) and breast cancer (BRCA) pathways to efficiently repair DNA DSBs. Indeed, these proteins have been established to form a complex by binding with GGN1.^25^, ^30^ More studies will help us to understand the involvement of GGNBP2 and GGN1 in the process of DNA DSB repair and to elucidate the nature of the interaction among GGNBP2, GGN1, FA and BRCA proteins to control meiotic prophase progression.

Given that GGNBP2 is a conserved zinc finger protein in human and mouse,^3^, ^4^, ^13^ it presumptively has similar functions in regulation of spermatogenesis in humans. A recent comprehensive analysis revealed that sperm counts continually and significantly declined over a 38‐year period in men from Western countries.^42^ Numerous reports have provided evidence that a decline in sperm count links to chronic exposure to environmental endocrine disrupting chemicals (EDCs).^43^, ^44^ Dioxins are a group of highly persistent and omnipresent EDCs.^45^ Decrease in sperm count is one of the most sensitive deteriorations of dioxin toxicity in both human and experimental studies.^45^, ^46^ However, the mechanism by which dioxins impair spermatogenesis remains elusive. The expression of GGNBP2 has been demonstrated to be regulated by dioxin and GGNBP2 plays critical roles in spermatocyte meiosis and spermatid differentiation.^4^, ^13^ Thereby, it would be worthwhile in future studies to investigate whether GGNBP2 is a factor that associates with dioxin‐induced reproductive toxicity by interfering with spermatogenesis.

In summary, the present data indicate that GGNBP2, together with GGN1, plays a regulatory role in germ cell meiosis. The results demonstrate that loss of *Ggnbp2* increases DNA DSBs and compromises DSB repair during male meiosis, suggesting that male infertility in *Ggnbp2KO* mice is partially attributed to the role of GGNBP2 in spermatocyte meiosis. Our findings provide a framework for further molecular studies of GGNBP2 in spermatogenesis.

## CONFLICT OF INTEREST

The authors declared no potential conflict of interests with respect to the research, authorship and/or publication of this article.

## AUTHOR CONTRIBUTIONS

ZML, HLW and KMG conceived and designed the experiments. KMG, YH, LYL, ZWL and XL performed the experiments. KMG, YH, JMZ and ZJL analysed the data. LC and ZJL contributed to reagents/materials/analysis tools. ZML and KMG wrote the manuscript.
